# Childhood Maltreatment Experience Influences Neural Response to Psychosocial Stress in Adults: An fMRI Study

**DOI:** 10.3389/fpsyg.2019.02961

**Published:** 2020-01-14

**Authors:** Xue Zhong, Qingsen Ming, Daifeng Dong, Xiaoqiang Sun, Chang Cheng, Ge Xiong, Chuting Li, Xiaocui Zhang, Shuqiao Yao

**Affiliations:** ^1^Medical Psychological Center, The Second Xiangya Hospital of Central South University, Changsha, China; ^2^Medical Psychological Institute, Central South University, Changsha, China; ^3^China National Clinical Research Center on Mental Disorders (Xiangya), Changsha, China; ^4^Department of Psychiatry, The First Affiliated Hospital of Soochow University, Suzhou, China

**Keywords:** childhood maltreatment, stress, cortisol, fMRI, sensitization

## Abstract

**Background:**

Childhood maltreatment is a strong risk factor for the development of depression in later life. However, the neurobiological mechanisms underlying this vulnerability are not well understood. As depression has been associated with dysfunction of the hypothalamic-pituitary-adrenal (HPA) axis and increased responsiveness to psychosocial stressors, we speculated that childhood maltreatment may lead to lasting alteration of the stress response system, thereby increasing the risk of depression. This study investigated the effects of childhood maltreatment on the stress response in healthy subjects while controlling for psychiatric condition.

**Methods:**

Forty-eight healthy young adults (24 females) with childhood maltreatment experience and 48 healthy controls (33 females) without such experience were administered the Montreal Imaging Stress Task during functional magnetic resonance imaging. Childhood maltreatment experience was assessed using the 28-item Childhood Trauma Questionnaire (CTQ). Between-group differences in subjective stress levels, whole brain activations and cortisol levels were assessed.

**Results:**

Relative to healthy control subjects, individuals exposed to childhood maltreatment exhibited higher subjective stress and cortisol levels. Neurofunctionally, participants with histories of childhood maltreatment displayed significantly increased activation in the dorsolateral prefrontal cortex (dlPFC), insula and precuneus, and decreased activation in ventromedial prefrontal cortex (vmPFC) relative to healthy controls during the psychosocial stress task. Activations in dlPFC and insula correlated with CTQ scores in the childhood maltreatment group.

**Conclusion:**

The results of this study show that childhood maltreatment induces lasting changes in brain function and HPA-axis responsiveness to stress. The observed abnormal activation in the dlPFC, insula and vmPFC and enhanced cortisol response are similar to those seen in individuals with depression. This dysfunction might serve as a diathesis that embeds latent vulnerability to psychiatric disorders, and this mechanism provides evidence supporting the stress sensitization model.

## Introduction

Childhood maltreatment, including physical abuse, emotional abuse, sexual abuse, physical neglect, and emotional neglect, is a potent predictor of poor mental health across the lifespan (McCrory et al., 2017). Individuals who have experienced childhood trauma have been found to have a four-fold increased risk of depression compared with those who have not experienced such trauma (Young et al., 1997). Although childhood adversity is clearly related to vulnerability to psychopathology, the specific mechanism underlying this relationship remains unclear.

Researches try to explore the relationship between early life stress and psychiatry disorders. According to the kindling hypothesis, life stressors may generate long-term vulnerabilities, thereby lowering the threshold of exposure required for episode recurrence, so that, over time, relatively minor stressors may trigger mood episodes (Post, 1992). As a severe psychosocial stressor, childhood maltreatment may increase vulnerability to ongoing life stress and lower the threshold required to trigger affective episodes (Post et al., 2001). Hammen et al. (2000) examined a number of childhood adversities and found a stress-sensitization effect in which women with higher levels of early adversity showed more depressive reactions to low levels of recent stressors compared with those with fewer adversities (Hammen et al., 2000). A growing body of evidence suggests that early childhood adversity has implications for stress reactivity and psychopathology throughout the entire life cycle (Post et al., 2001; Torrey, 2002; Heim et al., 2008; Belbasis et al., 2018).

One potential neurobiological mechanism of Post’s model is alterations in hypothalamic-pituitary-adrenal (HPA) axis regulation. Several studies support it. Engert et al. (2011) collected saliva from 58 healthy young participants at awakening and in the afternoon/evening, and found that participants with low parental care experiences exhibited increased cortisol awakening responses, increased afternoon/evening cortisol output, and increased depressive symptomatology. With the exception of basal cortisol status, individuals with childhood maltreatment histories have also shown abnormal activity of the HPA axis while performing psychosocial stress tasks, such as those included in the widely used Trier Social Stress Test (TSST; public speaking and oral mental arithmetic) (Kirschbaum et al., 1993). Heim et al. (2002) observed increased pituitary-adrenal responses during TSST tasks in adult women with histories of childhood abuse; these responses were particularly robust in abused women with depression or anxiety disorder (Heim et al., 2002). Study findings, however, are not consistent. In several studies, lower cortisol reactivity during the TSST has been observed in healthy adults (Carpenter et al., 2007, 2011) with histories of childhood trauma. Despite the inconsistency of findings, research suggests that childhood maltreatment has longstanding effects on the HPA axis.

Recent studies have employed functional magnetic resonance imaging (fMRI) to explore alterations in neurocognitive systems following childhood maltreatment. Structurally, childhood maltreatment has been associated with consistent alterations in the hippocampus, medial prefrontal cortex, and insula (Cohen et al., 2006; van Harmelen et al., 2010; Dannlowski et al., 2012). Alterations of brain function in maltreated children and adults include enhanced activation of fronto-limbic regions, such as the amygdala, dorsolateral prefrontal cortex (dlPFC), ventromedial prefrontal cortex (vmPFC), anterior cingulate cortex, and insula, in response to negative emotions (Dannlowski et al., 2012; Hart et al., 2018) and increased activation of the dorsomedial frontal gyrus and supplementary motor area during error processing (Lim et al., 2015). In addition, maltreatment has been associated consistently with enhanced amygdala response to threatening stimuli and diminished striatal response to anticipated reward (Dillon et al., 2009). Thus, these studies have demonstrated that childhood maltreatment is associated with altered function of a range of neurocognitive systems, including threat processing, reward processing, and emotion regulation. Several brain regions, such as the dlPFC, vmPFC, insula and hippocampus, are involved in the stress response and implicated in the pathophysiology of stress-related psychiatric problems (Wang et al., 2005; Pruessner et al., 2008; Dedovic et al., 2009; Ming et al., 2017). Alteration of the stress response system due to early adverse childhood experiences (Shapero et al., 2017) may underlie the increased risk of psychiatric disorders in affected individuals. Endocrinological studies have repeatedly shown that childhood maltreatment causes lasting changes in HPA axis responsiveness to stress and could thereby increase the risk of depression development. However, the potential effects on brain stress responses in adults with histories of childhood maltreatment have not been explored.

As the TSST requires speaking and alteration of experimental scenes, which causes head movement and hence is not optimal for functional imaging (Ming et al., 2017), no study has investigated responses under psychosocial stress in adults with histories of childhood maltreatment using such modalities. Other studies induced stress by a script which based on autobiographical memory (Seo et al., 2019; Zhai et al., 2019), which may have inherent limitations (Britton et al., 2005). In addition, the majority of studies describing maltreatment-related changes have been conducted with subjects already affected by major depressive disorder, anxiety, or posttraumatic stress disorder. Thus, it is difficult to infer whether alterations following early adversity are evident only in subjects who later develop psychiatric disorders, or whether these changes are detectable consequences of maltreatment in subjects with no psychiatric disorder history and accordingly constitute promising vulnerability markers. One study included healthy controls without current or lifetime psychiatric disorders which investigate the regional homogeneity and function connectivity under resting-state fMRI (Lu et al., 2017). To overcome these limitations, we applied the Montreal Imaging Stress Task (MIST), which comprises a series of computerized mental arithmetic tasks with an induced failure component and was developed to fit the constraints of the imaging environment (Dedovic et al., 2005), to investigate effects on physiological stressors and brain activation in healthy young subjects without psychiatric disorders.

In the present study, we aimed to explore HPA axis dysfunction and abnormal brain activity in a large sample of healthy, medication-naïve adults with and without childhood maltreatment histories who were carefully screened for psychiatric conditions. We employed the MIST to induce psychosocial stress. Based on the stress sensitization model and previous studies (Dedovic et al., 2009; Li et al., 2019), we hypothesized that healthy adults with childhood maltreatment histories would be more sensitive to psychosocial stress than would those with no such history. Specifically, we hypothesized that they would show increased cortisol levels and abnormal brain activity during the MIST.

## Materials and Methods

### Participants

In total, 100 healthy young subjects were recruited from two colleges and a Changsha community through posters and advertisements. Two experienced psychiatrists examined all subjects thoroughly and determined that they had no lifetime history of psychiatric disorder using the DSM-IV Structured Clinical Interview. Exclusion criteria were (1) prior DSM-IV-TR Axis I disorder; (2) history of psychotropic medication use or psychotherapy; (3) history of alcohol/substance abuse; and (4) diagnosed neurological disorder, structural brain abnormality, or contraindication for MRI. The Childhood Trauma Questionnaire (CTQ) was administered to assess maltreatment during childhood. This 28-item self-reported inventory screens for five types of childhood maltreatment: emotional, physical, and sexual abuse, and emotional and physical neglect. Responses are structured by a 5-point Likert scale ranging from “never true” to “very often true.” Childhood maltreatment is considered to have occurred when at least one subscale scale is above the threshold (emotional neglect >14, physical neglect >9, emotional abuse >12, physical abuse >9, sexual abuse >7) (Bernstein et al., 1994, 2003; Chaney et al., 2014). It has god reliability and validity among Chinese and is a good tool for child trauma assessment (He et al., 2019). Ultimately, 48 participants who had experienced childhood maltreatment (24 females) and 48 healthy controls (33 females) were recruited for the study. All subjects were right handed and of Han ancestry. They were compensated for their time and travel. The Ethics Committee of the Second Xiangya Hospital of Central South University, China, approved this study, and all participants provided written informed consent.

### The Montreal Imaging Stress Task

The MIST, a widely used stress paradigm adapted for fMRI, was administered to induce psychosocial stress using elements of uncontrollability and social evaluative threat (Dedovic et al., 2005). The MIST paradigm has a block design with three conditions (rest, control, and experimental). Under the rest condition, the participant’s baseline state was recorded for 30 s while the interface of the computer program remained on the screen with no task. Subjects were asked to keep their eyes open and not to press buttons until the next mental arithmetic task appeared. Under the control condition (90 s), brain activation related to a mental arithmetic task was recorded. This condition involves no time pressure; subjects were instructed to try to perform the task as accurately as possible. The average performance (rate of correct answers) is typically about 90% under this condition. Under the experimental condition (90 s), time pressure was induced with a time bar adapted to each subject’s performance in order to enforce a correct answer rate of about 50%. Subjects received “correct” or “incorrect” feedback after answering each math question, and a simulative performance bar at the top of the screen showed that their performance was below the correct rate of the “average subject,” which was artificially set to 80%. The sequence of conditions was repeated once during each measurement sequence, resulting in a total duration of 7 min. After each run, the investigator criticized the subject’s insufficient performance through headphones (for about 30 s), emphasizing the need for better performance to enhance the subject’s perceived stress. We investigated the contrasts experimental minus control to assess the effect of stress.

### Image Acquisition

Functional magnetic resonance imaging data were acquired using a 3.0-T Siemens Magnetom Skyra scanner (Siemens Healthineers, Erlangen, Germany). Blood oxygen level–dependent data were acquired with an echo planar imaging sequence (repetition time, 2 s; echo time, 30 ms; flip angle, 80°; field of view, 256 mm × 256 mm; 64 × 64 matrix; 32 slices; 4-mm slice thickness with 1-mm intervals).

### Psychological and Physiological Measures

All participants completed the Beck Depression Inventory-II (BDI), a validated self-reported scale of depressive symptoms (Beck et al., 1996). Levels of subjective stress were assessed immediately before and after each imaging run using a visual analog scale ranging from 0 (absence of stress) to 10 (maximal stress). Stress level changes were calculated by subtracting pre-stress from post-stress values. Saliva samples were collected with a Salivette tube (Sarstedt, Nümbrecht, Germany) upon participant arrival (Cort1), after a 30-min rest (Cort2), upon entering the scanner (Cort3), during anatomical imaging (Cort4), after MIST runs 1–3 (Cort5–7), and upon leaving the scanner (Cort8). We used cortisol increases from Cort4 (baseline, before the stress task) to Cort8 (highest, after the task) as summary measures of cortisol response, as this approach does not depend on the accurate timing of repeated cortisol measurement. To control for circadian fluctuation, the scans were carried out between 2:00 and 5:00 pm. Cortisol concentrations were detected using a human cortisol ELISA Kit (Bio-Swamp, Shanghai, China).

### Statistical Analysis

Statistical analyses of behavioral data were performed using SPSS 18.0. For the behavioral analysis, one-sample *t* tests and chi-squared tests were used in all subjects and groups. Repeated-measures analyses of variance were used to measure the main effects of stress and group on cortisol concentrations and subjective stress levels.

The Statistical Parametric Mapping 12 software (Wellcome Department of Imaging Neuroscience, London, United Kingdom) was used for imaging data analysis. Images were realigned. After realignment, images were co-registered to the high resolution EPI. All scans were normalized to standard space, using the EPI template, with the parameters derived from the high resolution EPI and applied to the functional time series. We also used T1 image to normalization. Results were in Supplementary Materials (Supplementary Tables S1, S2 and Supplementary Figure S1). And data were smoothed with a 6-mm full-width at half-maximum Gaussian kernel. Individuals whose scans showed excessive head movement (translation >1.5 mm or rotation >1.5°) were excluded from the analyses. For each participant, imaging data were modeled using a block design. We computed t maps of experimental versus control conditions for each subject. At the first level, six movement parameters were included as regressors. At the second level, the independent-sample *t* test was applied to detect between-group differences in the whole brain with age, gender and education scores as covariates. Statistical significance was set with an initial voxel-level threshold of *p* < 0.001 uncorrected, to which a FWE-correction at the cluster-level was applied (*p* < 0.05). Marsbar^1^ was used to extract beta values from a 6-mm-radius sphere around the peak of activation that differed between groups for analysis of correlation between neural activation, childhood trauma scores and cortisol responses. Correlations were assessed using SPSS 18.0.

## Results

### Participant Characteristics and Stress Responses

Data from two control subject and two subjects who had experienced childhood maltreatment were excluded due to excessive head movement, resulting in a final cohort of 48 subjects who had experienced childhood maltreatment (CM) and 48 controls (Non-CM). The groups did not differ in terms of gender, age, or years of education. BDI and CTQ subscale scores were significantly higher in the childhood maltreatment group than in the control group (see Table 1).

**TABLE 1:** Demographic characteristics of people experienced childhood maltreatment and healthy comparison subjects.

**Characteristic**	**CM (*N* = 48)**	**Non-CM (*N* = 48)**	**χ*^2^/t***	***p***
Gender (M/F)	24/24	15/33	χ^2^ = 3.498	0.096
Age, years	22.67 ± 411	22.42 ± 2.77	*t* = 0.481	0.632
Education, years	14.08 ± 2.30	14.85 ± 1.49	*t* = -1.511	0.134
BDI score	5.08 ± 4.61	2.80 ± 3.01	*t* = 2.864	0.005
**CTQ scores**				
Total	42.75 ± 9.11	33.32 ± 4.84	*t* = 6.332	<0.001
Emotional abuse	7.52 ± 2.91	6.28 ± 1.68	*t* = 2.562	0.012
Physical abuse	5.94 ± 1.66	5.56 ± 0.99	*t* = 1.341	0.183
Sexual abuse	6.23 ± 2.57	5.13 ± 0.44	*t* = 2.933	0.004
Emotional neglect	12.23 ± 4.77	9.15 ± 3.08	*t* = 3.758	<0.001
Physical neglect	10.83 ± 2.34	7.21 ± 1.52	*t* = 9.017	<0.001

*M/F, number of males/number of females; BDI, Beck Depression Inventory; CTQ, Childhood Trauma Questionnaire. Means (all variables except gender) are reported with standard deviations.*

**FIGURE 1:**
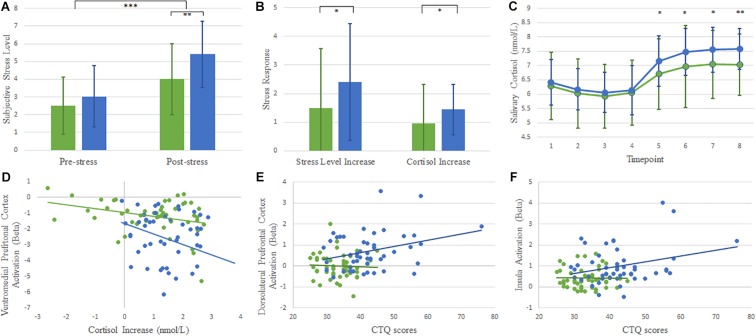
Graph **(A)** shows that the subjective stress levels were greater after performing the MIST task than before the task in both groups. The childhood maltreatment group experienced higher stress levels than healthy control group. Graph **(B)** shows that the childhood maltreatment group exhibited greater cortisol during the MIST. Graph **(C)** shows the salivary cortisol concentrations throughout the experiment: participant’s arrival (1), after a 30-min rest (2), upon entering the scanner (3), during anatomical imaging (4), after Montreal Imaging Stress Task runs 1–3(5–7), and upon leaving the scanner (8). Graph **(D)** shows a scatterplot of the significant negative correlations between ventromedial prefrontal cortex and cortisol responses in both groups. Graphs **(E,F)** show scatterplots of correlations between childhood trauma scores and activation in dorsolateral prefrontal cortex and insula. Error bars represent standard deviations. ^∗^*p* < 0.05, ^∗∗^*p* < 0.01, ^∗∗∗^*p* < 0.001.

Subjective stress increased significantly after the MIST, with significant main effects of stress (*F*_1_,_94_ = 86.144, *p* < 0.001) and group (*F*_1_,_94_ = 10.132, *p* = 0.002), and group-by-stress interaction (*F*_1_,_94_ = 4.555, *p* = 0.035). Subjects with childhood maltreatment histories reported higher stress levels than did control subjects (Figure 1A).

Baseline (Cort1–4) cortisol levels did not differ significantly between groups. Cortisol levels increased significantly after the MIST in both groups. A repeated-measures general linear model of cortisol concentrations revealed a main effect of time (*F*_7_,_658_ = 40.594, *p* < 0.001), with a distinct increase after task commencement. A main effect of group (*F*_1_,_94_ = 7.207, *p* = 0.009) was detected, due to greater responses in subjects with histories of childhood maltreatment compared with control subjects (Figure 1C). Intra-MIST cortisol increases were greater among subjects who had experienced childhood maltreatment than among control subjects (*t* = 2.030, *p* = 0.045; Figure 1B). These cortisol and self-reported stress results indicated that stress was successfully induced across groups, and individuals with histories of childhood maltreatment experienced greater reactivity to stress.

### fMRI Results

**FIGURE 2:**
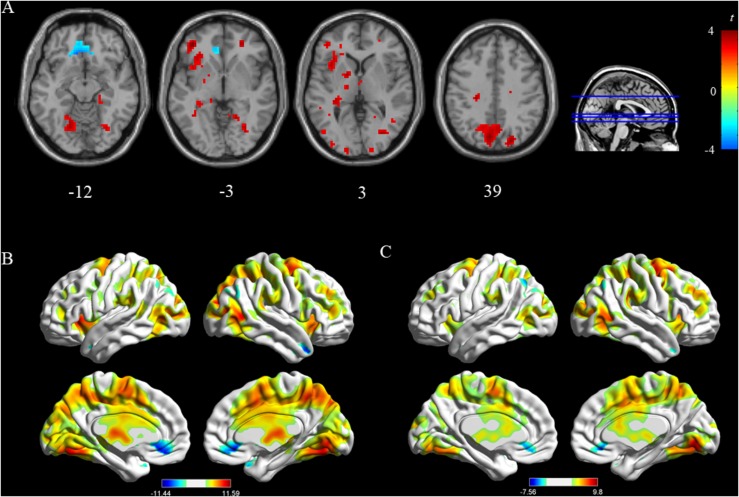
Panel **(A)** shows comparison between the childhood maltreatment group and healthy control group. Panels **(B,C)** shows activated regions of the stress in childhood maltreatment group and healthy control group (*p* < 0.05, family wise error rate corrected).

In the childhood maltreatment group, we observed significant deactivation of the vmPFC, angular and increased activation in the occipital lobe (fusiform, lingual gyrus), middle frontal gyrus, and insula (Figure 2B). Among healthy controls, deactivation was detected in the vmPFC. Activation was located mainly in the lingual gyrus and frontal superior gyrus (all *p* < 0.05 [FWE corrected]; Table 2 and Figure 2C).

**TABLE 2:** Stress-induced activity changes in childhood maltreatment group and healthy control group.

**Group**	**Effect**	**Region**	**Hemisphere**	**MNI**			***t***	***p***
				***x***	***y***	***z***		
CM	Activation	Fusiform	Right	27	−66	−6	11.59	<0.001
		Lingual gyrus	Right	18	−69	−6	10.79	<0.001
		Insula	Left	−36	15	3	9.84	<0.001
			Right	36	21	−6	8.80	<0.001
		Middle frontal gyrus	Left	−30	45	15	8.68	<0.001
	Deactivation	Medial frontal gyrus	Right	9	30	−12	11.44	<0.001
			Left	−9	30	−12	11.05	<0.001
		Superior temporal gyrus	Right	39	18	−33	10.38	<0.001
			Left	−36	18	−30	6.97	<0.001
		Angular	Right	54	−69	36	7.36	<0.001
		Parahippocampal gyrus	Right	24	−9	−18	5.16	<0.001
Non-CM	Activation	Lingual gyrus	Right	9	−75	−6	9.8	<0.001
		Fusiform	Right	27	−66	−6	8.79	<0.001
		Frontal superior gyrus	right	27	−6	57	8.61	<0.001
		Insula	Left	−30	24	0	5.62	<0.001
	Deactivation	Medial frontal gyrus	Right	9	30	−12	7.56	<0.001
			Left	−12	30	−12	5.82	<0.001

*MNI, Montreal Neurological Institute coordinates. *p* < 0.05 family wise error rate-corrected at the cluster level.*

To identify brain activation in response to the stressful task, we subtracted control from experimental values for all subjects. Two-sample *t* tests showed that the childhood maltreatment group exhibited significantly increased activity in dlPFC, insula, and precuneus, and decreased activity in vmPFC compared with controls without childhood maltreatment experiences (all *p* < 0.05 [FWE corrected]; Table 3 and Figure 2A).

**TABLE 3:** Comparison of stress-related activation in childhood maltreatment group and healthy control group.

**Contrast and region**	**Hemisphere**	**MNI**			***t***	***p***
		***x***	***y***	***z***		
**CM > Non-CM**						
Dorsolateral prefrontal cortex	Left	−39	45	−3	4.82	<0.001
Insula	Left	−36	15	3	4.11	<0.001
Precuneus	Left	−6	−75	39	4.38	<0.001
**CM < Non-CM**						
Ventromedial prefrontal cortex	Left	−9	30	−12	4.96	0.005

**p* < 0.05 family wise error rate-corrected at the cluster level.*

### Correlations

We assessed correlations among regional activation, cortisol responses and CTQ scores. Cortisol responses correlated with activation in the vmPFC in both groups (CM: *r* = −0.311, *p* = 0.32; Non-CM: *r* = −0.316, *p* = 0.029, Figure 1D). CTQ scores correlated with activation in the dlPFC and insula in childhood maltreatment group (*r*_dlPFC_ = 0.304, *p* = 0.035; *r*_insula_ = 0.289, *p* = 0.046; Figures 1E,F), indicating that these regions were linked to childhood maltreatment experience.

## Discussion

To our knowledge, this study is the first to investigate the effects of childhood trauma on cortisol and brain reactivity using a psychosocial stress task in healthy people. Using the MIST, we successfully induced stress in participants that was apparent on subjective, physiological, and neural levels. Participants who had experienced childhood maltreatment exhibited more subjective stress than did controls. As hypothesized, at the endocrine level, young healthy people with histories of childhood maltreatment showed increased cortisol responses relative to healthy controls. At the neurofunctional level, participants with histories of childhood maltreatment showed increased activation in the dlPFC, insula and precuneus reduced activation in the vmPFC compared with controls. Post’s (Post, 2007, 2016) model implicates early adversity as a mechanism through which individuals become sensitized to future proximal stressors. Our findings seem to support this model at the endocrine and neurofunctional levels, and provide evidence of the long-lasting effects of early development experiences, including vulnerability to depression and other stress-related disorders.

Higher subjective stress and heightened cortisol responsivity to the MIST were observed in both groups, indicating that this experimental paradigm successfully elicited stress. Our findings are concordant with previous findings of significantly increased cortisol responsiveness to psychosocial stressors among individuals who have experienced childhood maltreatment relative to those who have not (Heim et al., 2000; Pesonen et al., 2010), and indicate that severe stress in early life is associated with persistent sensitization of the pituitary-adrenal stress response. MDD has frequently been linked to dysregulation of the HPA axis, characterized by hyperactivity indicated by the hypersecretion of cortisol (Vreeburg et al., 2009). The HPA-axis activity patterns of maltreated individuals facing psychosocial stress are similar to those of patients with MDD. In line with the kindling hypothesis that prior psychosocial stress acts primarily as a “trigger” of depression onset, abnormal HPA-axis reactivity to stress in subjects with histories of childhood maltreatment may reflect biological vulnerability to the development of stress-related psychiatric disorders.

Compared with healthy controls, individuals with childhood maltreatment experience exhibited decreased activity in vmPFC during the MIST. The medial prefrontal cortex plays a major role in controlling of behavior, regulating cognitive and emotional processes, and is implicated in stress regulation through extensive interconnections with other cortical and subcortical regions (Dedovic et al., 2009; McCrory et al., 2012). Previous studies also found that vmPFC deficits are associated with altered regulation of emotions (Morese et al., 2016; Lo Gerfo et al., 2019). Decreased activation in vmPFC may indicate the lack of ability to manage the emotion regulation and then may constitute a potential risk factor when facing stressors in abused group. Hypoactivation in vmPFC indicates that individuals with childhood maltreatment experience exhibited more sensitivity to proximal stressors, which speculates that dysfunction in vmPFC as a consequence of childhood maltreatment may result to stress sensitization to future stress. Furthermore, we also found that the extent of vmPFC deactivation correlated with the extent of elevation of the cortisol response, suggesting a relationship with HPA axis activation. The medial prefrontal cortex is known to inhibit the paraventricular nucleus of the hypothalamus, which regulates pituitary and adrenal release of cortisol. Reduced activation of the vmPFC leads to attenuated inhibition of cortisol release (Diorio et al., 1993). Similar relationships have been reported previously; decreased activity in the prefrontal cortex has been associated with increased cortisol secretion in response to a psychological stress task, supporting a central role of the medial prefrontal cortex in HPA-axis stress-response regulation (Ming et al., 2017). Taken together, this finding suggests that impaired processing in stress systems is a marker of latent vulnerability and sheds light on the mechanisms implicated in the pathogenesis of psychiatric disorders.

Individuals with childhood maltreatment experience showed increased activation relative to healthy controls without adversity history in dlPFC and insula during stress context. Structural and functional abnormalities of the dlPFC have been shown to have significance for cognitive and emotional dysregulations and are commonly observed in individuals with early adversity experience (Pechtel and Pizzagalli, 2011). The insula is interconnected with prefrontal cortical and limbic structure and implicated in cognitive control, interoceptive awareness and emotion process (Craig, 2002). As a part of salience network, insula plays an important role in detecting novel and salient environmental stimuli and trigger appropriate control signals to regulate homeostatic state (Seeley et al., 2007; Menon and Uddin, 2010; Zhang et al., 2016). Increased activation in these regions may suggest individuals with childhood maltreatment experience may have excessive activations when facing psychosocial stress. Enhanced reactivity to stressors may be a common adaptive response to early maltreatment. The adaption may be beneficial to maintain sustained vigilance in early adversity, but it may also constitute a latent neurobiological risk factor of psychopathology vulnerability. Notably, we found significantly correlations between activation of dlPFC and insula and CTQ scores in childhood maltreatment group only. These findings support the kindling model that early adversity leads to epigenetic modifications that serve to increase sensitivity to later stress. Taken together, the present study suggests that early childhood experiences lead to dlPFC and insula sensitivity, which sensitizes individuals to subsequent proximal stress and increasing risk for stress-related disorders.

Hyperactivation of the precuneus was detected in the childhood maltreatment group compared with the control group. The precuneus is a major component of the default mode network and has been implicated in high-level cognitive functions, including episodic memory, self-related processing, and aspects of consciousness (Cavanna and Trimble, 2006; Zhong et al., 2016). Previous studies also showed that precuneus dysfunction correlated with depressive symptoms, such as somatic complaints and negative bias in interpreting bodily feedback (Cavanna, 2007). Hence, abnormal precuneus activation in individuals with childhood maltreatment experience may favor self-focused processing and enhance awareness of negative social evaluations. Overall, this finding suggests that precuneus hyperactivity is a key factor in maladaptive stress-related negative emotionality.

The current study has several limitations. First, we did not control for the menstrual cycle or collect data on female participants’ hormonal status (which may affect stress responses) (Goldstein et al., 2010) during fMRI scans. Hence, we could not differentiate the potential influences of gonadal and other hormones on neural responses to stress, which need to be studied in future research. Second, the inclusion of a group of abused young people without psychiatric disorders would have enabled more robust identification of abuse-specific deficits; however, such a “pure” abused group would not be representative of the general abused population, as severe abuse is typically associated with psychiatric comorbidity (Dannlowski et al., 2012). More studies, especially those with longitudinal designs, are needed to shed light on these issues.

## Conclusion

This study is the first to investigate neural psychosocial stress processing in individuals who have experienced childhood maltreatment but have no psychiatric disorder. Compared with controls without early adversity, these individuals with early life stress exhibited increased cortisol levels, reduced activation in vmPFC, and increased activation in the dlPFC, insula and precuneus in response to psychosocial stress. Childhood maltreatment may induce lasting changes in brain and HPA-axis function, making individuals who have experienced early adversity more sensitive when facing psychosocial stressors. This mechanism may increase the risk of depression development and provides evidence supporting the stress sensitization model.

## Data Availability Statement

The datasets generated for this study are available on request to the corresponding author.

## Ethics Statement

The studies involving human participants were reviewed and approved by the Ethics Committee of the Second Xiangya Hospital of Central South University. The patients/participants provided their written informed consent to participate in this study. Written informed consent was obtained from the individual(s) for the publication of any potentially identifiable images or data included in this manuscript.

## Author Contributions

SY supervised the study. XuZ performed the analysis and wrote the manuscript. QM, DD, XS, CC, GX, CL, and XiZ helped to collect data and carry out the research. All authors revised and approved the version to be published.

## Conflict of Interest

The authors declare that the research was conducted in the absence of any commercial or financial relationships that could be construed as a potential conflict of interest.

## Footnotes

1 http://marsbar.sourceforge.net/

## References

[B1] Beck A. T. Steer R. A. Brown G. (1996). *Manual for the Beck Depression Inventory-II.* San Antonio, TX: Psychological Corporation.

[B2] Belbasis L. Kohler C. A. Stefanis N. Stubbs B. van Os J. Vieta E. (2018). Risk factors and peripheral biomarkers for schizophrenia spectrum disorders: an umbrella review of meta-analyses. *Acta Psychiatr. Scand.* 137 88–97. 10.1111/a.12847 29288491

[B3] Bernstein D. P. Fink L. Handelsman L. Foote J. Lovejoy M. Wenzel K. (1994). Initial reliability and validity of a new retrospective measure of child abuse and neglect. *Am. J. Psychiatry* 151 1132–1136. 10.1176/ajp.151.8.1132 8037246

[B4] Bernstein D. P. Stein J. A. Newcomb M. D. Walker E. Pogge D. Ahluvalia T. (2003). Development and validation of a brief screening version of the childhood trauma questionnaire. *Child Abuse Negl.* 27 169–190. 10.1016/s0145-2134(02)00541-0 12615092

[B5] Britton J. C. Phan K. L. Taylor S. F. Fig L. M. Liberzon I. (2005). Corticolimbic blood flow in posttraumatic stress disorder during script-driven imagery. *Biol. Psychiatry* 57 832–840. 10.1016/j.biopsych.2004.12.025 15820703

[B6] Carpenter L. L. Carvalho J. P. Tyrka A. R. Wier L. M. Mello A. F. Mello M. F. (2007). Decreased adrenocorticotropic hormone and cortisol responses to stress in healthy adults reporting significant childhood maltreatment. *Biol. Psychiatry* 62 1080–1087. 10.1016/j.biopsych.2007.05.002 17662255 PMC2094109

[B7] Carpenter L. L. Shattuck T. T. Tyrka A. R. Geracioti T. D. Price L. H. (2011). Effect of childhood physical abuse on cortisol stress response. *Psychopharmacology* 214 367–375. 10.1007/s00213-010-2007-2004 20838776 PMC3580170

[B8] Cavanna A. E. (2007). The precuneus and consciousness. *CNS Spectr.* 12 545–552. 10.1017/s1092852900021295 17603406

[B9] Cavanna A. E. Trimble M. R. (2006). The precuneus: a review of its functional anatomy and behavioural correlates. *Brain* 129(Pt 3), 564–583. 10.1093/brain/awl004 16399806

[B10] Chaney A. Carballedo A. Amico F. Fagan A. Skokauskas N. Meaney J. (2014). Effect of childhood maltreatment on brain structure in adult patients with major depressive disorder and healthy participants. *J. Psychiatry Neurosci.* 39 50–59. 10.1503/jpn.120208 23900024 PMC3868665

[B11] Cohen R. A. Grieve S. Hoth K. F. Paul R. H. Sweet L. Tate D. (2006). Early life stress and morphometry of the adult anterior cingulate cortex and caudate nuclei. *Biol. Psychiatry* 59 975–982. 10.1016/j.biopsych.2005.12.016 16616722

[B12] Craig A. D. (2002). How do you feel? Interoception: the sense of the physiological condition of the body. *Nat. Rev. Neurosci.* 3 655–666. 10.1038/nrn894 12154366

[B13] Dannlowski U. Stuhrmann A. Beutelmann V. Zwanzger P. Lenzen T. Grotegerd D. (2012). Limbic scars: long-term consequences of childhood maltreatment revealed by functional and structural magnetic resonance imaging. *Biol. Psychiatry* 71 286–293. 10.1016/j.biopsych.2011.10.021 22112927

[B14] Dedovic K. D’Aguiar C. Pruessner J. C. (2009). What stress does to your brain: a review of neuroimaging studies. *Can. J. Psychiatry* 54 6–15. 10.1177/070674370905400104 19175975

[B15] Dedovic K. Renwick R. Mahani N. K. Engert V. Lupien S. J. Pruessner J. C. (2005). The Montreal Imaging Stress Task: using functional imaging to investigate the effects of perceiving and processing psychosocial stress in the human brain. *J. Psychiatry Neurosci.* 30 319–325. 16151536 PMC1197276

[B16] Dillon D. G. Holmes A. J. Birk J. L. Brooks N. Lyons-Ruth K. Pizzagalli D. A. (2009). Childhood adversity is associated with left basal ganglia dysfunction during reward anticipation in adulthood. *Biol. Psychiatry* 66 206–213. 10.1016/j.biopsych.2009.02.019 19358974 PMC2883459

[B17] Diorio D. Viau V. Meaney M. J. (1993). The role of the medial prefrontal cortex (cingulate gyrus) in the regulation of hypothalamic-pituitary-adrenal responses to stress. *J. Neurosci.* 13 3839–3847. 10.1523/jneurosci.13-09-03839.1993 8396170 PMC6576467

[B18] Engert V. Efanov S. I. Dedovic K. Dagher A. Pruessner J. C. (2011). Increased cortisol awakening response and afternoon/evening cortisol output in healthy young adults with low early life parental care. *Psychopharmacology* 214 261–268. 10.1007/s00213-010-1918-1914 20596856

[B19] Goldstein J. M. Jerram M. Abbs B. Whitfield-Gabrieli S. Makris N. (2010). Sex differences in stress response circuitry activation dependent on female hormonal cycle. *J. Neurosci.* 30 431–438. 10.1523/JNEUROSCI.3021-09.2010 20071507 PMC2827936

[B20] Hammen C. Henry R. Daley S. E. (2000). Depression and sensitization to stressors among young women as a function of childhood adversity. *J. Consult. Clin. Psychol.* 68 782–787. 10.1037//0022-006x.68.5.782 11068964

[B21] Hart H. Lim L. Mehta M. A. Simmons A. Mirza K. A. H. Rubia K. (2018). Altered fear processing in adolescents with a history of severe childhood maltreatment: an fMRI study. *Psychol. Med.* 48 1092–1101. 10.1017/S0033291716003585 29429419 PMC6088776

[B22] He J. Zhong X. Gao Y. Xiong G. Yao S. (2019). Psychometric properties of the Chinese version of the Childhood Trauma Questionnaire-Short Form (CTQ-SF) among undergraduates and depressive patients. *Child Abuse Negl.* 91 102–108. 10.1016/j.chiabu.2019.03.009 30856597

[B23] Heim C. Newport D. J. Heit S. Graham Y. P. Wilcox M. Bonsall R. (2000). Pituitary-adrenal and autonomic responses to stress in women after sexual and physical abuse in childhood. *JAMA* 284 592–597. 10918705 10.1001/jama.284.5.592

[B24] Heim C. Newport D. J. Mletzko T. Miller A. H. Nemeroff C. B. (2008). The link between childhood trauma and depression: insights from HPA axis studies in humans. *Psychoneuroendocrinology* 33 693–710. 10.1016/j.psyneuen.2008.03.008 18602762

[B25] Heim C. Newport D. J. Wagner D. Wilcox M. M. Miller A. H. Nemeroff C. B. (2002). The role of early adverse experience and adulthood stress in the prediction of neuroendocrine stress reactivity in women: a multiple regression analysis. *Depress. Anxiety* 15 117–125. 10.1002/da.10015 12001180

[B26] Kirschbaum C. Pirke K. M. Hellhammer D. H. (1993). The ‘Trier Social Stress Test’–a tool for investigating psychobiological stress responses in a laboratory setting. *Neuropsychobiology* 28 76–81. 10.1159/000119004 8255414

[B27] Li C. Sun X. Dong D. Zhong X. Wang X. Yao S. (2019). Effect of corticotropin-releasing hormone receptor1 gene variation on psychosocial stress reaction via the dorsal anterior cingulate cortex in healthy adults. *Brain Res.* 1707 1–7. 10.1016/j.brainres.2018.11.020 30447186

[B28] Lim L. Hart H. Mehta M. A. Simmons A. Mirza K. Rubia K. (2015). Neural Correlates of Error Processing in Young People With a History of Severe Childhood Abuse: an fMRI Study. *Am. J. Psychiatry* 172 892–900. 10.1176/appi.ajp.2015.14081042 25882324

[B29] Lo Gerfo E. Gallucci A. Morese R. Vergallito A. Ottone S. Ponzano F. (2019). The role of ventromedial prefrontal cortex and temporo-parietal junction in third-party punishment behavior. *Neuroimage* 200 501–510. 10.1016/j.neuroimage.2019.06.047 31233906

[B30] Lu S. Gao W. Wei Z. Wang D. Hu S. Huang M. (2017). Intrinsic brain abnormalities in young healthy adults with childhood trauma: a resting-state functional magnetic resonance imaging study of regional homogeneity and functional connectivity. *Aust. N. Z. J. Psychiatry* 51 614–623. 10.1177/0004867416671415 27694638

[B31] McCrory E. De Brito S. A. Viding E. (2012). The link between child abuse and psychopathology: a review of neurobiological and genetic research. *J. R. Soc. Med.* 105 151–156. 10.1258/jrsm.2011.110222 22532655 PMC3343716

[B32] McCrory E. J. Gerin M. I. Viding E. (2017). Annual Research Review: childhood maltreatment, latent vulnerability and the shift to preventative psychiatry - the contribution of functional brain imaging. *J. Child Psychol. Psychiatry* 58 338–357. 10.1111/jcpp.12713 28295339 PMC6849838

[B33] Menon V. Uddin L. Q. (2010). Saliency, switching, attention and control: a network model of insula function. *Brain Struct. Funct.* 214 655–667. 10.1007/s00429-010-0262-260 20512370 PMC2899886

[B34] Ming Q. Zhong X. Zhang X. Pu W. Dong D. Jiang Y. (2017). State-Independent and Dependent Neural Responses to Psychosocial Stress in Current and Remitted Depression. *Am. J. Psychiatry* 174 971–979. 10.1176/appi.ajp.2017.16080974 28618857

[B35] Morese R. Rabellino D. Sambataro F. Perussia F. Valentini M. C. Bara B. G. (2016). Group Membership Modulates the Neural Circuitry Underlying Third Party Punishment. *PLoS One* 11:e0166357. 10.1371/journal.pone.0166357 27835675 PMC5106004

[B36] Pechtel P. Pizzagalli D. A. (2011). Effects of early life stress on cognitive and affective function: an integrated review of human literature. *Psychopharmacology* 214 55–70. 10.1007/s00213-010-2009-2002 20865251 PMC3050094

[B37] Pesonen A. K. Raikkonen K. Feldt K. Heinonen K. Osmond C. Phillips D. I. (2010). Childhood separation experience predicts HPA axis hormonal responses in late adulthood: a natural experiment of World War II. *Psychoneuroendocrinology* 35 758–767. 10.1016/j.psyneuen.2009.10.017 19963324

[B38] Post R. M. (1992). Transduction of psychosocial stress into the neurobiology of recurrent affective disorder. *Am. J. Psychiatry* 149 999–1010. 10.1176/ajp.149.8.999 1353322

[B39] Post R. M. (2007). Kindling and sensitization as models for affective episode recurrence, cyclicity, and tolerance phenomena. *Neurosci. Biobehav. Rev.* 31 858–873. 10.1016/j.neubiorev.2007.04.003 17555817

[B40] Post R. M. (2016). Epigenetic basis of sensitization to stress, affective episodes, and stimulants: implications for illness progression and prevention. *Bipolar Disord.* 18 315–324. 10.1111/bdi.12401 27346321

[B41] Post R. M. Leverich G. S. Xing G. Weiss R. B. (2001). Developmental vulnerabilities to the onset and course of bipolar disorder. *Dev. Psychopathol.* 13 581–598. 10.1017/s0954579401003091 11523849

[B42] Pruessner J. C. Dedovic K. Khalili-Mahani N. Engert V. Pruessner M. Buss C. (2008). Deactivation of the limbic system during acute psychosocial stress: evidence from positron emission tomography and functional magnetic resonance imaging studies. *Biol. Psychiatry* 63 234–240. 10.1016/j.biopsych.2007.04.041 17686466

[B43] Seeley W. W. Menon V. Schatzberg A. F. Keller J. Glover G. H. Kenna H. (2007). Dissociable intrinsic connectivity networks for salience processing and executive control. *J. Neurosci.* 27 2349–2356. 10.1523/JNEUROSCI.5587-06.2007 17329432 PMC2680293

[B44] Seo D. Rabinowitz A. G. Douglas R. J. Sinha R. (2019). Limbic response to stress linking life trauma and hypothalamus-pituitary-adrenal axis function. *Psychoneuroendocrinology* 99 38–46. 10.1016/j.psyneuen.2018.08.023 30172968 PMC6436805

[B45] Shapero B. G. Weiss R. B. Burke T. A. Boland E. M. Abramson L. Y. Alloy L. B. (2017). Kindling of Life Stress in Bipolar Disorder: effects of Early Adversity. *Behav. Ther.* 48 322–334. 10.1016/j.beth.2016.12.003 28390496 PMC5385846

[B46] Torrey E. F. (2002). Early physical and sexual abuse associated with an adverse course of bipolar illness. *Biol. Psychiatry* 52 843–845. 12372656 10.1016/s0006-3223(02)01495-6

[B47] van Harmelen A. L. van Tol M. J. van der Wee N. J. Veltman D. J. Aleman A. Spinhoven P. (2010). Reduced medial prefrontal cortex volume in adults reporting childhood emotional maltreatment. *Biol. Psychiatry* 68 832–838. 10.1016/j.biopsych.2010.06.011 20692648

[B48] Vreeburg S. A. Hoogendijk W. J. van Pelt J. Derijk R. H. Verhagen J. C. van Dyck R. (2009). Major depressive disorder and hypothalamic-pituitary-adrenal axis activity: results from a large cohort study. *Arch. Gen. Psychiatry* 66 617–626. 10.1001/archgenpsychiatry.2009.50 19487626

[B49] Wang J. Rao H. Wetmore G. S. Furlan P. M. Korczykowski M. Dinges D. F. (2005). Perfusion functional MRI reveals cerebral blood flow pattern under psychological stress. *Proc. Natl. Acad. Sci. U.S.A.* 102 17804–17809. 10.1073/pnas.0503082102 16306271 PMC1292988

[B50] Young E. A. Abelson J. L. Curtis G. C. Nesse R. M. (1997). Childhood adversity and vulnerability to mood and anxiety disorders. *Depress. Anxiety* 5 66–72. 10.1002/(sici)1520-6394(1997)5:2<66::aid-da2=3.3.co;2-x 9262936

[B51] Zhai Z. W. Yip S. W. Lacadie C. M. Sinha R. Mayes L. C. Potenza M. N. (2019). Childhood trauma moderates inhibitory control and anterior cingulate cortex activation during stress. *Neuroimage* 185 111–118. 10.1016/j.neuroimage.2018.10.049 30342975 PMC6392043

[B52] Zhang X. Di X. Lei H. Yang J. Xiao J. Wang X. (2016). Imbalanced spontaneous brain activity in orbitofrontal-insular circuits in individuals with cognitive vulnerability to depression. *J. Affect. Disord.* 198 56–63. 10.1016/j.jad.2016.03.001 27011360

[B53] Zhong X. Pu W. Yao S. (2016). Functional alterations of fronto-limbic circuit and default mode network systems in first-episode, drug-naive patients with major depressive disorder: a meta-analysis of resting-state fMRI data. *J. Affect. Disord.* 206 280–286. 10.1016/j.jad.2016.09.005 27639862

