# Supplementary material for: Childhood Maltreatment Experience Influences Neural Response to Psychosocial Stress in Adults: An fMRI Study
Source: Front Psychol. 2020 Jan 14;10:2961. doi: 10.3389/fpsyg.2019.02961 (PMC6971063; doi:10.3389/fpsyg.2019.02961)
Supplement: Supplementary file 1 [file Table_1.docx]

**Supplementary Materials**

**Method**

**In this imaging pre-processing pipeline, we used the functional to structural co-registration. Results are the same as co-registered to the high resolution EPI pre-processing pipeline.**

**STable 1.** Stress-induced activity changes in childhood maltreatment group and healthy control group.

| **Group** | **Effect** | **Region** | **Hemisphere** | **MNI** | | | ***t*** | ***p*** |
| --- | --- | --- | --- | --- | --- | --- | --- | --- |
|  |  |  |  | **x** | **y** | **z** |  |  |
| **CM** | Activation | Middle frontal gyrus | Right | 39 | -6 | 54 | 10.51 | <0.001 |
|  |  | Lingual gyrus | Right | 21 | -69 | -6 | 10.08 | <0.001 |
|  |  | Fusiform | Right | 30 | -69 | -12 | 9.94 | <0.001 |
|  |  | Insula | Left | -39 | 15 | 3 | 8.13 | <0.001 |
|  | Deactivation | Angular | Right | 54 | -69 | 27 | 10.37 | <0.001 |
|  |  | Medial frontal gyrus | Left | -9 | 33 | -6 | 8.36 | <0.001 |
|  |  |  | Right | 9 | 30 | -3 | 6.97 | <0.001 |
|  |  | Superior temporal gyrus | Right | 45 | 15 | -24 | 7.78 | <0.001 |
| **Non-CM** | Activation | Lingual gyrus | Right | 9 | -75 | -6 | 9.34 | <0.001 |
|  |  | Fusiform | Right | 27 | -66 | -3 | 9.08 | <0.001 |
|  |  | Frontal superior gyrus | Right | 18 | 12 | 60 | 7.95 | <0.001 |
|  |  | Insula | Left | -30 | 27 | 0 | 6.18 | <0.001 |
|  | Deactivation | Superior temporal gyrus | Right | 45 | 12 | -33 | 7.72 | 0.002 |
|  |  | Medial frontal gyrus | Right | 3 | 39 | -12 | 7.13 | <0.001 |
|  |  |  | Left | -6 | 39 | -12 | 6.88 | <0.001 |

Note: MNI, Montreal Neurological Institute coordinates. p<0.05 family-wise error rate-corrected at the cluster level.

**STable 2.** Comparison of stress-related activation in childhood maltreatment group and healthy control group.

| **Contrast and Region** | **Hemisphere** | **MNI** | | | ***t*** | ***p*** |
| --- | --- | --- | --- | --- | --- | --- |
|  |  | **x** | **y** | **z** |  |  |
| **CM>Non-CM** |  |  |  |  |  |  |
| Dorsolateral prefrontal cortex | Left | -42 | 45 | -3 | 4.81 | 0.016 |
| Precuneus | Left | -6 | -72 | 42 | 4.70 | <0.001 |
| Insula | Left | -36 | 21 | 3 | 4.33 | 0.022 |
| **CM <Non-CM** |  |  |  |  |  |  |
| Ventromedial prefrontal cortex | Left | -9 | 33 | -6 | 4.46 | 0.007 |

Note: p<0.05 family-wise error rate-corrected at the cluster level.

**SFigure1** Brain activation of group comparison and CM, Non-CM group.


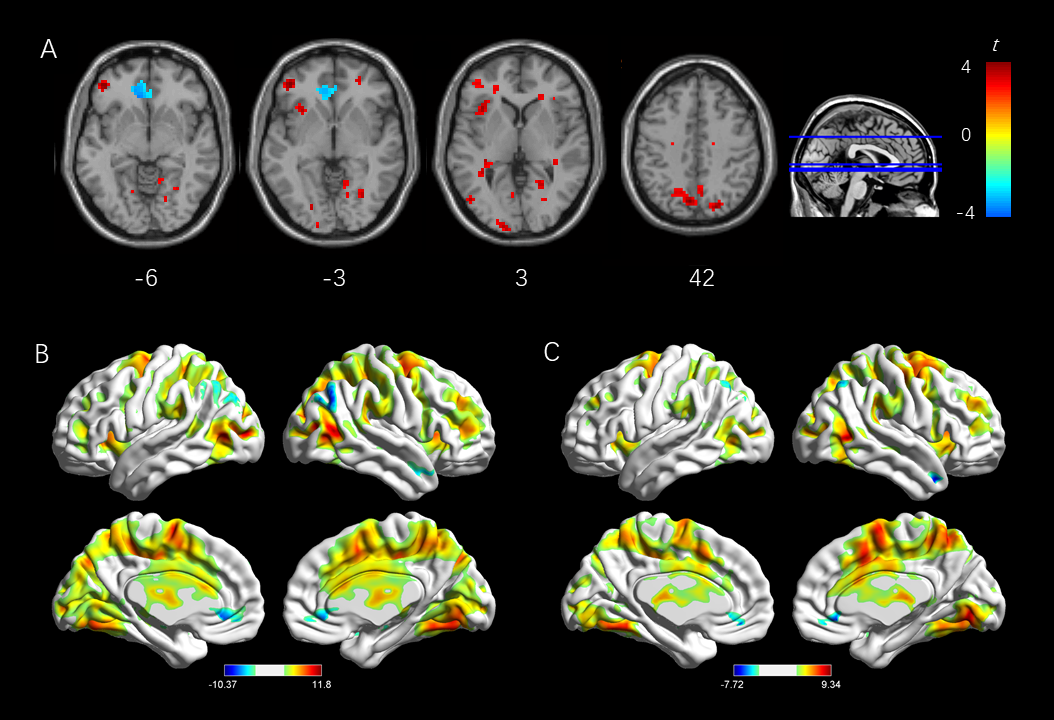


Note: Panel A shows comparison between the childhood maltreatment group and healthy control group. Graph B and C shows activated regions of the stress in childhood maltreatment group and healthy control group (p<0.05, family-wise error rate corrected).
